# Secreted Thrombospondin-1 Regulates Macrophage Interleukin-1β Production and Activation through CD47

**DOI:** 10.1038/srep19684

**Published:** 2016-01-27

**Authors:** Erica V. Stein, Thomas W. Miller, Kelly Ivins-O’Keefe, Sukhbir Kaur, David D. Roberts

**Affiliations:** 1 Laboratory of Pathology, Center for Cancer Research, National Cancer Institute, National Institutes of Health, Bethesda, MD 20892-1500, USA; 2 Microbiology and Immunology Program of the Institute for Biomedical Sciences, Departments of Microbiology, Immunology and Tropical Medicine, George Washington University, Washington, DC, 20037, USA

## Abstract

Thrombospondin-1 regulates inflammation by engaging several cell surface receptors and by modulating activities of other secreted factors. We have uncovered a novel role of thrombospondin-1 in modulating production and activation of the proinflammatory cytokine IL-1β by human and murine macrophages. Physiological concentrations of thrombospondin-1 limit the induction by lipopolysaccharide of IL-1β mRNA and total protein production by human macrophages. This inhibition can be explained by the ability of thrombospondin-1 to disrupt the interaction between CD47 and CD14, thereby limiting activation of NFκB/AP-1 by lipopolysaccharide. Only the CD47-binding domain of thrombospondin-1 exhibits this activity. In contrast, CD47, CD36, and integrin-binding domains of thrombospondin-1 independently enhance the inflammasome-dependent maturation of IL-1β in human THP-1 monocyte-derived macrophages. Correspondingly, mouse bone marrow-derived macrophages that lack either thrombospondin-1 or CD47 exhibit diminished induction of mature IL-1β in response to lipopolysaccharide. Lack of CD47 also limits lipopolysaccharide induction of IL-1β, NLRP3, and caspase-1 mRNAs. These data demonstrate that thrombospondin-1 exerts CD47-dependent and -independent pro-and anti-inflammatory effects on the IL-1β pathway. Therefore, thrombospondin-1 and its receptor CD47 may be useful targets for limiting the pro-inflammatory effects of lipopolysaccharide and for treating endotoxemia.

Therapeutics that modulate immune responses have potential applications ranging from enhancing anti-tumor immunity to preventing the excessive inflammatory responses associated with autoimmunity and septic shock^1^. Therapeutic antibodies that block specific immune inhibitory pathways such as that mediated by PD1 binding to PD-L1 have yielded durable remissions of several cancers^2^.

The interaction between CD47 and its counter-receptor signal regulatory protein-α (SIRPα) controls another major inhibitory pathway that limits innate and adaptive tumor immunity^3^,^4^. Two antibodies that block the interaction of CD47 on tumor cells with SIRPα on macrophages have entered Phase 1 human clinical trials (NCT02216409, NCT02367196, NCT02488811). These CD47 antibodies enhance phagocytosis of tumor cells by macrophages *in vitro* and promote clearance of human tumor xenografts in *Nod/SCID* mice that express a mutant SIRPα capable of binding human CD47 with high affinity^3^,^5^,^6^.

To assess the potential utility of CD47-targeted therapeutics in cancer, one must consider additional physiological and pathophysiological functions of CD47 that could result in detrimental side effects^7^. Based on the known role of CD47 in limiting phagocytic clearance of red blood cells, anemia is one expected side effect and has been observed in primates^8^,^9^,^10^. Mice lacking CD47 are also known to be more susceptible to some microbial pathogens^11^, and we recently reported increased susceptibility of CD47-deficient mice to *Candida albicans*, which is a frequent cause of fatal disseminated candidemia in immunocompromised cancer patients^12^. The increased virulence of *C. albicans* in *cd47*^−/−^ mice was associated with alterations in innate immunity and adaptive T cell immunity. Macrophage recruitment into infected organs of *cd47*^−/−^ mice was altered, but phagocytosis and killing of *C. albicans* by macrophages was unchanged. This deficit in macrophage recruitment may result from loss of SIRPα signaling in macrophages^13^.

In addition to engaging SIRPα and SIRPγ as a counter-receptor, CD47 is high affinity signaling receptor for thrombospondin-1 (TSP1)^7^,^14^. TSP1 circulates at low levels in plasma and is expressed by many cell types including innate immune cells in response to injury or stress^15^,^16^,^17^,^18^. The absence of TSP1 or CD47 confers increased resistance of cells and tissues to a variety of stresses, and TSP1 signaling through CD47 limits cellular responses to stress through regulation of metabolism, protective autophagy, nitric oxide, and c-Myc signaling^7^,^19^,^20^.

Previous studies indicate that TSP1 has context-specific pro- and anti-inflammatory functions^21^,^22^,^23^. Endogenous TSP1 enhances the pathogenesis of systemic *C. albicans* infections in mice despite enhancing macrophage activation and PMN recruitment^24^. The presence of TSP1 also exacerbates surgical and non-surgical peritonitis, but in this context, decreases phagocytic killing, thereby increasing bacterial load during infection^25^. Ligation of the TSP1 receptor CD47 with a synthetic TSP1 peptide or blocking antibody to TSP1, in the presence of *Staphylococcus aureus* Cowan I protein, significantly inhibited dendritic cells maturation and cytokine production, including the amount of secreted IL-12 and TNFα^26^.

The complex TSP1 effects on inflammation are consistent with the existence of multiple TSP1 receptors and binding partners (reviewed in^27^). Macrophages express at least three known TSP1 receptors, and each mediates distinct functions. Binding of the N-terminal domain of TSP1 to α6β1 integrin promotes M1 macrophage differentiation or recruitment into tumors and enhances phorbol ester-induced superoxide production and target tumor cell killing by macrophages^28^. Interaction of the central thrombospondin type 1 repeats of TSP1 with the receptor CD36 stimulates macrophage IL-10 expression^29^ and contributes to activation of TLR4 signaling^30^. TSP1 binding to CD47 inhibits macrophage release of IL-12^31^ and may promote macrophage recruitment into injured vascular tissue^17^. Ligation with a CD47-blocking antibody, in the presence of infectious *E. coli,* overcame inhibitory TSP1 signaling that prevented the maturation of immature dendritic cells into mature DCs that produce inflammatory cytokines^32^. This inhibitory CD47 signal was not affected by knockdown of SIRPα. However, treatment of monocytes and dendritic cells with a CD47-binding peptide also induced cell death^33^. Infiltration of CD68^+^ macrophages into liver tissue after ischemia/reperfusion injury was similarly reduced in *thbs1*^−/−^ and *cd47*^−/−^ mice, suggesting that TSP1 signaling through CD47 controls macrophage recruitment or activation^34^. TSP1 signaling through CD47 also limits the expression of stem cell transcription factors. Consequently, the ability of induced multipotent stem cells from *cd47* null mice to be reprogrammed into Mac2^+^ macrophages in the presence of M-CSF was increased^35^.

Based on prior reports that CD47 associates with lipid rafts containing CD14^36^ and that TSP1 binding dissociates CD47 from its lateral associations with two other membrane receptors^37^,^38^, we hypothesized that TSP1 binding to CD47 could inhibit CD14-dependent LPS-stimulated transcription of pro-IL-1β mRNA. We report here that expression of CD47 promotes IL-1β transcription and pro-IL-1β maturation. TSP1 ligation of CD47 inhibits LPS-induced IL-1β mRNA expression by limiting CD14-CD47 association, but induces maturation of pro-IL-1β.

## Results

### Decreased caspase-1 and IL-1β responses in the absence of CD47 or TSP1

Active IL-1β is produced in a two-step cascade. The first signal (e.g. LPS) promotes transcription of pro-IL-1β mRNA. LPS binds and induces dimerization and internalization of TLR4 with the help of the co-receptor CD14^39^. This up-regulates transcription factors, including NFκB and AP-1, which induce transcription and translation of inactive pro-IL-1β^40^,^41^. The second signal (e.g. ATP) activates the zymogen pro-IL-1β to its mature form by caspase-1-mediated cleavage^42^.

**Figure 1:**
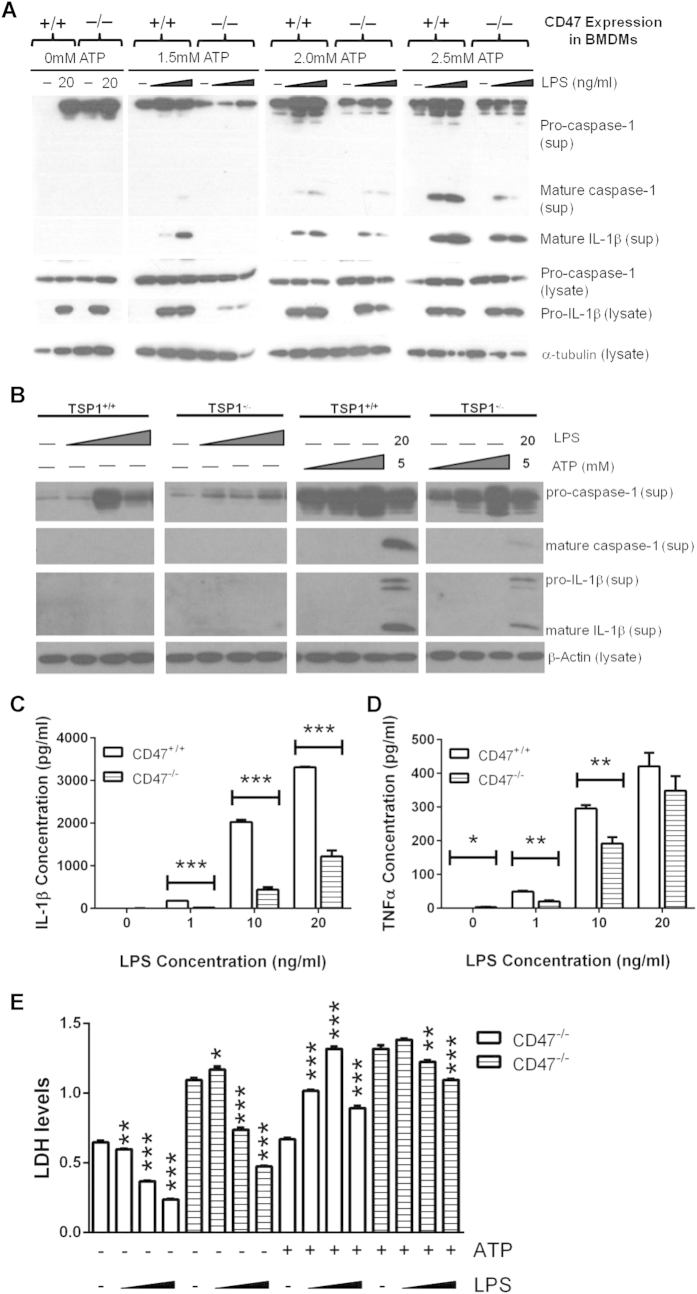
Wild type BMDMs produce more mature caspase-1 and IL-1β than *cd47*^−/−^ or *thbs1*^−/−^ bone marrow derived macrophages when treated with LPS and ATP. (**A**) Adherent wild type and *cd47*^−/−^ BMDMs were treated with medium or LPS (10 ng/ml or 20 ng/ml) for 3 h. 2.5 mM ATP was added at the concentrations indicated for the last 30 min. (**B**) Wild type and *thbs*^−/−^ BMDMs were cultured in medium or LPS (10 ng/ml, 20 ng/ml, or 50 ng/ml) for 4.5 h. Then either medium or ATP (1 mM, 5 mM, 10 mM) was added to both sets of culture for an additional hour. sup = conditioned medium from the well following experiments; lysate = cell lysate. (**C–E**) BMDMs were stimulated with indicated concentration of LPS for 3 h and 2.5 mM ATP was added to every sample for the last 30 min of incubation. ELISAs were used to quantify total secreted IL-1β (**C**) and TNFα (**D**). (**E**) Samples were tested for release of lactate dehydrogenase. In panels (**C,D**) significance was calculated using unpaired Student’s t-test. In panel E significance was calculated using one-way parametric ANOVA. When data significance is indicated, “*” means p < 0.05, “**” means p < 0.01, and “***” means p < 0.005. Data shown is a representative of 3 independent experiments performed.

Wild type and *cd47*^−/−^ bone marrow-derived macrophages (BMDMs) were used to compare mature and immature caspase-1 and IL-1β levels induced by the canonical inflammasome activation pathway. The pathogen-associated molecular pattern (PAMP) LPS was used to trigger signal one through TLR4, and ATP was used as a purinergic stimulator of signal two. Both cell types produced secreted pro-caspase-1 in all stimulation conditions, but only after combined LPS and ATP simulation was mature caspase-1 and mature IL-1β secreted. Wild type cells treated with 10 or 20 ng/ml LPS in combination with all tested concentrations of ATP released more mature caspase-1 and IL-1β into the medium compared to *cd47*^−/−^ BMDMs (Fig. 1A). Although basal differences in pro-caspase-1 and pro-IL-1β were not significant when measured in twelve independent experiments, we observed a trend towards less intracellular pro-IL-1β induced by LPS in *cd47*^−/−^ BMDMs (data not shown). This trend is represented in the presented experiment. BMDMs prepared from *thbs1*^−/−^ mice also released less mature IL-1β and mature caspase-1 after stimulation with 20 ng/ml LPS and 5 mM ATP (Fig. 1B).

Levels of pro-IL-1β released into the medium from cells treated with LPS plus ATP also tended to be lower in *thbs1*^−/−^ and *cd47*^−/−^ versus wild type BMDMs (Fig. 1A,B). An ELISA that recognizes both immature and mature IL-1β was used to quantify total secreted levels of this protein. At each concentration of LPS and in the presence of 2.5 mM ATP, *cd47*^−/−^ BMDMs released significantly less total IL-1β (p ≤ 0.005, Fig. 1C). *cd47*^−/−^ BMDMs also secreted significantly less of the inflammatory cytokine TNFα at 1 ng/ml LPS and 10 ng/ml LPS (Fig. 1D, p ≤ 0.001), and levels trended lower at 20 ng/ml LPS concentrations but did not achieve significance.

Caspase-1 can induce a form of pro-inflammatory programmed cell death known as pyroptosis^43^. We measured cell death to determine if CD47-driven IL-1β activation was a result of pyroptosis. All cells were treated with 20 ng/ml LPS for 3 hours either with or without ATP added for the final 30 minutes. In the absence of ATP, treatment of wild type or *cd47*^−/−^ cells with 10 or 20 ng/ml LPS resulted in a significant decrease in cell death (Fig. 1E, p ≤ 0.005). Similarly, significantly less LDH was released by the *cd47*^−/−^ cells when ATP was added in the last 30 minutes of LPS treatment (p ≤ 0.01). Therefore, in the *cd47*^−/−^ *cells,* LPS-induced IL-1β production was independent of pyroptosis. On contrast, treatment of wild type BMDMs with any concentration of LPS and ATP significantly increased cell death (p ≤ 0.005). These findings indicate that *cd47*^−/−^ cells are resistant to caspase-1 dependent pyroptosis compared to wild type BMDM.

### LPS and TSP1 synergize to activate IL-1β

**Figure 2:**
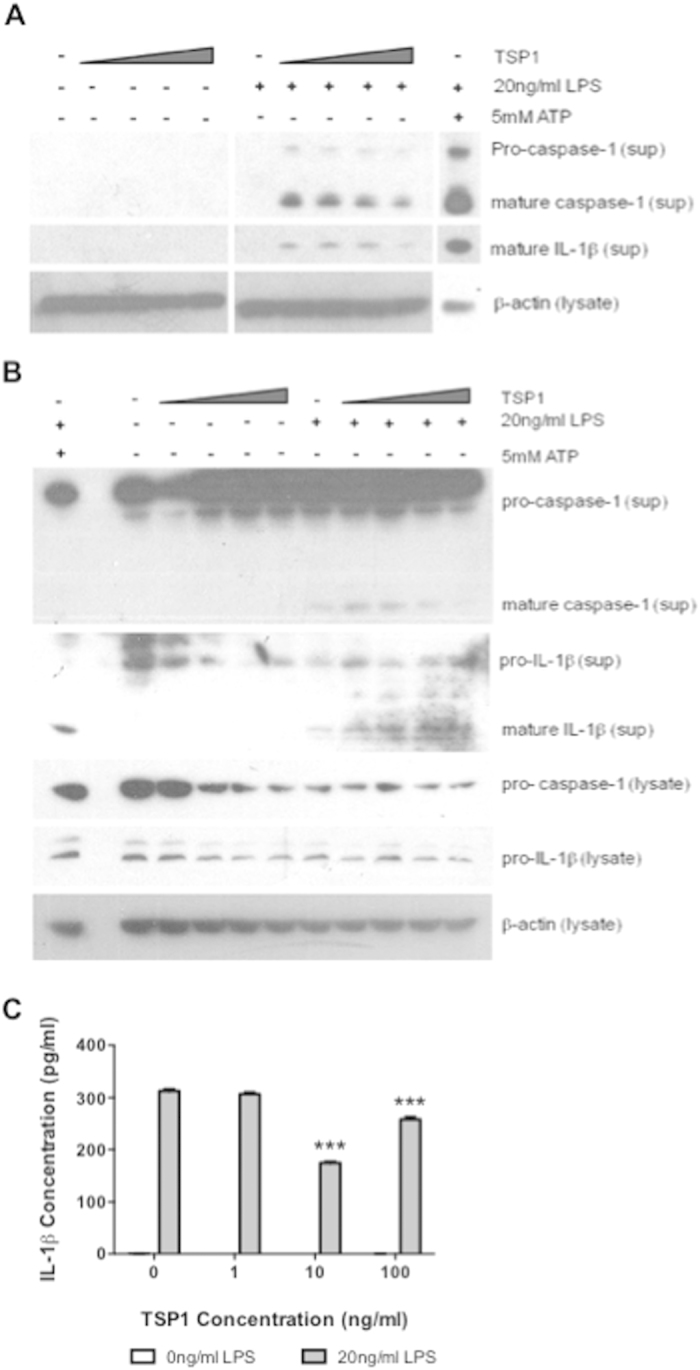
Soluble TSP1 in the presence of LPS causes IL-1β maturation. (**A**) Differentiated THP-1 were incubated with soluble TSP1 (10, 30, 100, 300 ng/ml). (**B**) Primary monocyte derived macrophages were incubated with soluble TSP1 (0.1, 1, 10, 100 ng/ml). After 15-20 min either medium or 20 ng/ml LPS was added for an additional 3 h. Medium or 5 mM ATP was added in the final 30 min where indicated. Supernatant (sup) and cell lysates (lys) from each experiment were collected and analyzed by western blot for caspase-1, IL-1β and β-actin. (**C**) Supernatant was analyzed for total secreted human IL-1β using ELISA. In panel B significance was calculated using one-way parametric ANOVA. When data significance is indicated “*” means p < 0.05, “**” means p < 0.01, and “***” means p < 0.005. Data shown is a representative of 3 independent experiments performed.

The preceding data suggested that TSP1 signaling through its receptor CD47 has a positive effect on the maturation of pro-IL-1β. Therefore, we stimulated macrophages derived from the human THP-1 monocytic cell line with soluble human TSP1 to test if LPS and TSP1 are sufficient to stimulate secretion of mature caspase-1 and IL-1β. Treatment of THP-1-derived macrophages with 30–300 ng/ml of soluble TSP1 without LPS produced no detectable secretion of pro-caspase-1, mature caspase-1, or mature IL-1β (Fig. 2A). However, supernatants from THP-1 cells stimulated with LPS following TSP1 pre-treatment showed increased secretion of mature caspase-1 and mature IL-1β at 10 ng/ml TSP1 but less so at higher TSP1 concentrations. These results were confirmed in primary monocyte-derived macrophages (Fig. 2B). To further assess this apparent biphasic response we used an IL-1β ELISA to quantify total secreted levels of IL-1β. Soluble TSP1 did not increase the total released IL-1β levels stimulated by LPS in macrophages derived from THP-1 cells, but total IL-1β release significantly decreased (p ≤ 0.005) when cells were treated with 0.1, 10, and 100 ng/ml TSP1 prior to LPS stimulation (Fig. 2C). Although macrophages may express substantial levels of TSP1 in response to inflammatory stimuli^17^, the data in Fig. 2 indicates that exogenous TSP1 has a negative effect on signal one in the absence of a signal two agonist.

### CD47 increases LPS-induced IL-1β, NLRP3, and caspase-1 mRNA levels

**Figure 3:**
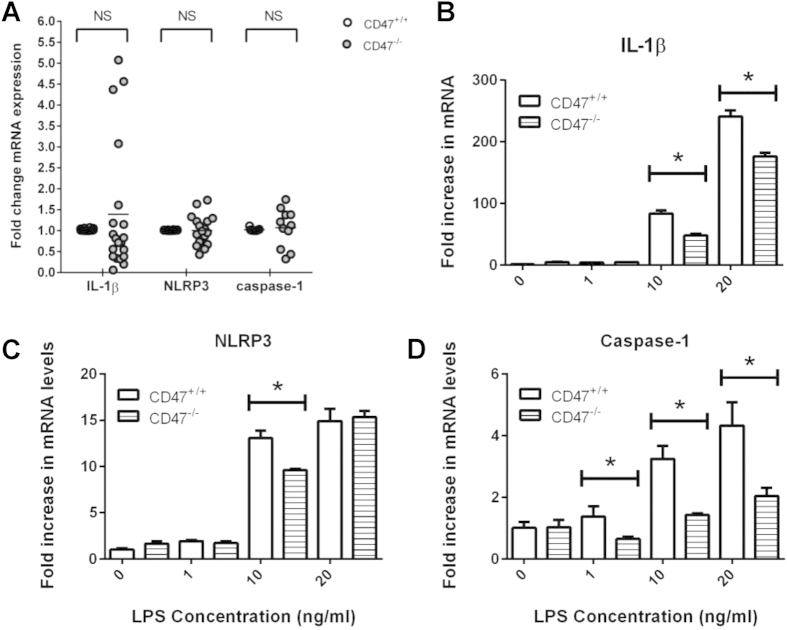
Wild type BMDMs have significantly more caspase-1 and IL-1β mRNA than *cd47*^−/−^ bone marrow derived macrophages after LPS stimulation. (**A**) Graph depicts basal levels of IL-1β, NLRP3, and caspase-1 in wild type and *cd47*^−/−^ mRNAs following twenty separate experiments. (**B–D**) Cells were stimulated with LPS as indicated for 3 h. 2.5 mM ATP was added to wells for the final 30 min. Levels of IL-1β, NLRP3, and caspase-1 mRNA were measured using quantitative real-time PCR. In panel (**A**) significance was calculated using two-way ANOVA. In panel (**B**) significance was calculated using unpaired Student’s t-test. When data significance is indicated “*” means p < 0.05, “**” means p < 0.01, and “***” means p < 0.005. Data shown is a representative of 3 independent experiments performed.

These differences between mature IL-1β assessed by blotting and total secreted IL-1β responses assessed by ELISA suggested that CD47 signaling modulates signal one stimulated by LPS, which induces the transcription of several inflammasome-related genes^44^. Therefore, we evaluated wild type and *cd47*^−/−^ BMDMs for IL-1β, NLRP3, and caspase-1 mRNA levels after LPS treatment. Although basal IL-1β mRNA levels exhibited significant mouse-to-mouse variability, mean basal mRNA levels for IL-1β, NLRP3, and caspase-1 did not differ between wild type and *cd47*^−/−^ BMDMs (Fig. 3A). However, treatment with 10–20 ng/ml LPS induced significantly more IL-1β mRNA in wild type BMDMs (p ≤ 0.05, Fig. 3B), more NLRP3 mRNA after 10 ng/ml LPS treatment (p ≤ 0.05, Fig. 3C) and more caspase-1 mRNA after any LPS treatment (p ≤ 0.05, Fig. 3D). Therefore, in the absence of added exogenous TSP1, CD47 positively regulates LPS-induced transcription of inflammasome-related genes including IL-1β.

### Soluble TSP1 decreases IL-1β and caspase-1 mRNA induction by LPS stimulation in human macrophages via CD47

**Figure 4:**
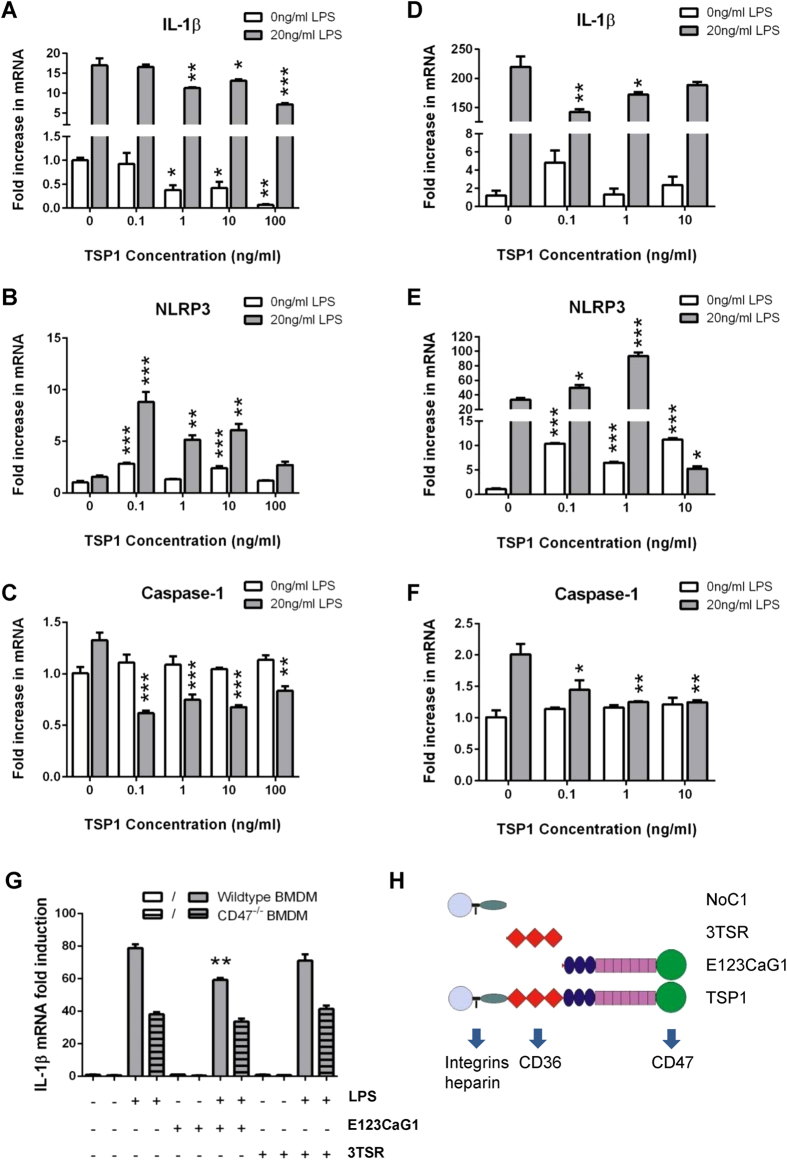
In human cells TSP1 inhibits IL-1β and caspase-1 mRNA, but not NLRP3 induced in the presence of LPS. (**A–C**) Differentiated THP-1 cells were cultured with soluble human TSP1 and after 15–20 min were either left untreated or stimulated with 20 ng/ml LPS for an additional 3 h. (**D–F**) Primary human macrophages were treated with TSP1 (as shown) or OptiMEM alone for 15–20 min, and then 20 ng/ml LPS was added for 3 h. (**A,D**) IL-1β, (**B,E**) NLRP3, and (**C,F**) caspase-1 mRNA expression was analyzed by quantitative real-time PCR. (**G**) Bone marrow from wild type and *cd47*^−/−^ mice was taken and differentiated into BMDMs. Cells were pre-treated with of recombinant domains of TSP1 (22 pM of E123CaG1 or 3TSR) or OptiMEM alone for 15–20 min, and then 20 ng/ml LPS or medium alone was added for 3 h. The level of IL-1β mRNA quantified using real-time PCR. (**H**) Recombinant domains of TSP1 and their relevant receptor specificities. For panels A through G significance was calculated using one-way parametric ANOVA. When data significance is indicated “*” means p < 0.05, “**” means p < 0.01, and “***” means p < 0.005. Data shown is a representative of 3 independent experiments performed.

Because TSP1 can signal through several receptors that are expressed on macrophages, it was not clear that the CD47-dependent stimulation of pro-IL-1β, NLRP3, and pro-caspase-1 mRNA induction involves TSP1. THP-1 and primary monocyte-derived macrophages were primed with physiological levels of TSP1 comparable to those circulating in human plasma^45^,^46^ and left either untreated or stimulated with 20 ng/ml LPS. Interestingly, pro-IL-1β mRNA levels were significantly decreased in THP-1 cells treated with 1-100 ng/ml TSP1, independent of LPS addition (p ≤ 0.05, Fig. 4A). Similar results were obtained for primary macrophages treated with 0.1–1 ng/ml TSP1 prior to LPS stimulation (p ≤ 0.05, Fig. 4D). Additionally, concentrations of pro-caspase-1 were significantly lower in both THP-1 cells and primary macrophages when primed with any concentration of TSP1 prior to LPS stimulation (p ≤ 0.05, Fig. 4C,F). In contrast, THP-1 and primary macrophages, primed with 0.1–10 ng/ml TSP1 and stimulated with 20 ng/ml LPS, had significantly enhanced NLRP3 mRNA concentrations (p ≤ 0.05, Fig. 4B,E). Typically, LPS was not necessary to induce NLRP3 levels if cells had already been primed with TSP1. Therefore, these data show that TSP1 inhibits the induction of pro-IL-1β and pro-caspase-1 mRNA, but not the induction of NLRP3 mRNA. The negative effects of TSP1 on pro-caspase-1 and pro-IL-1β mRNA levels may represent an antagonism of basal CD47 signaling or may be mediated by a different TSP1 receptor.

To further examine the mechanism by which TSP1 inhibits LPS-induced pro-IL-1β mRNA levels, we treated wild type or *cd47*^−/−^ murine BMDMs with two recombinant domains of TSP1 with known receptor specificity. E123CaG1 constitutes the C-terminal domain of TSP1 that interacts specifically with CD47^47^ (Fig. 4H). In wild type BMDMs, E123CaG1 significantly inhibited pro-IL-1β mRNA induction when used at the same molar concentration where intact TSP1 inhibited the LPS-stimulated signal one response (p ≤ 0.01, Fig. 4G). This inhibitory activity of E123CaG1 required CD47 because no inhibition of LPS-induced pro-IL-1β mRNA expression was observed in *cd47*^−/−^ BMDMs. TSP1 signaling, through CD36 was previously reported to mediate TLR4-dependent pro-inflammatory signaling^30^. Therefore, a recombinant domain of TSP1 containing its three type 1 repeats (3TSR), which interact with the receptor CD36 and activate latent TGFβ1, was used as a control. Treatment of wild type or *cd47*^−/−^ BMDMs with 3TSR, in combination with LPS, did not inhibit LPS-induced pro-IL-1β mRNA induction. Therefore, TSP1 signaling through CD47 is necessary and sufficient to inhibit signal one of the inflammasome pathway activated by LPS.

### CD47 binding to CD14 and CD14/TLR4 signaling are inhibited by TSP1

**Figure 5:**
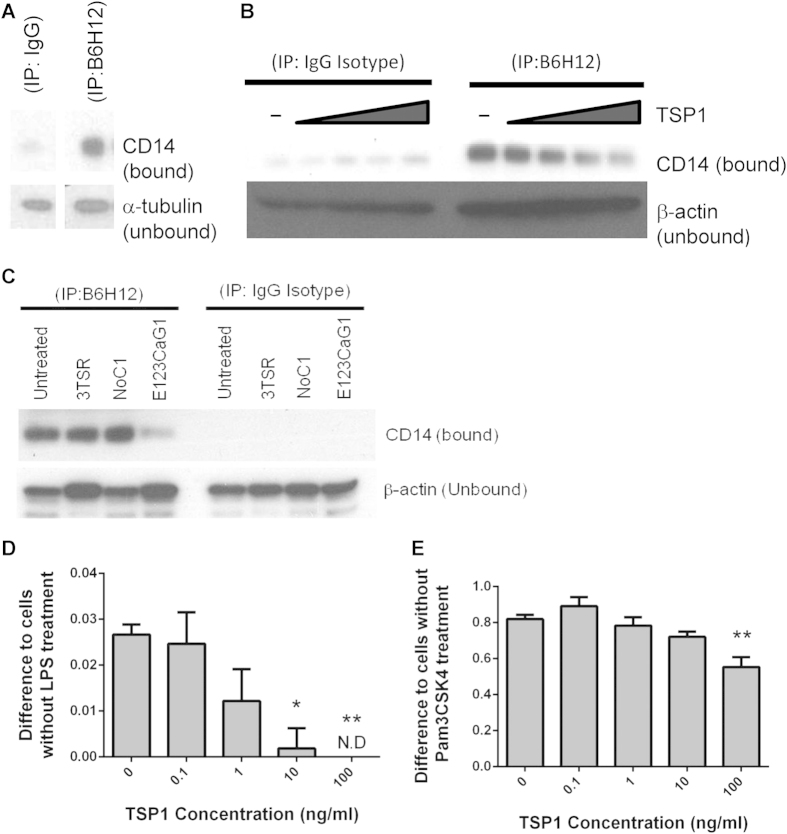
CD47 associates with CD14 and is dissociated by TSP1. Differentiated THP-1 cells were incubated for 15 min in the presence of (**A**) 22 pM soluble human TSP1 or (**C**) 22 pM of recombinant 3TSR, NoC1, or E123CaG1. (**B**) Primary human monocyte derived macrophages were incubated with increasing concentrations of human soluble TSP1 (0.1, 1, 10, 100 ng/ml) for 15 min. CD14 was precipitated using or IgG-k anti-mouse isotype control antibody or mouse anti-human CD47 (B6H12) monoclonal antibody. Bound = protein that precipitated with respective antibodies; Unbound = lysate after precipitation by the beads. (**D–E**) THP1-XBlue cells were differentiated stimulated with indicated concentrations of TSP1 for 15–20 min. Cells were left untreated or (**D**) treated with 20 ng/ml LPS or (**E**) 100 ng/ml Pam3CSK4 for 3 h. Sterile QuantiBlue was added to quantify NFκB/AP-1 with the inducible reporter (SEAP) system, and cells were incubated for an additional 2 h. In panels D and E significance was calculated using one-way parametric ANOVA. When data significance is indicated “**” means p < 0.01. ND = not detected. Data shown is a representative of 3 independent experiments performed.

LPS signaling through TLR4 requires prior binding of LPS to the chaperone CD14 and transfer to MD-2. CD14 laterally associates with several proteins in the plasma membrane, including CD47, and LPS was reported to induce dissociation of CD47 from CD14^36^. However, the function of CD47 in TLR4/CD14 signal transduction was not examined. Because CD47 is known to associate laterally with other signaling receptors, including VEGFR2, and TSP1 binding to CD47 inhibits VEGFR2 signaling by dissociating CD47^38^, we hypothesized that interaction of CD47 with the CD14 complex might similarly modulate signal one. We first confirmed physical interactions between CD14 and CD47 in THP-1 cells. CD14 was prominently detected by western blotting in an anti-CD47 immunoprecipitate, but was absent in the control IgG immunoprecipitates (Fig. 5A). CD14 similarly co-precipitated with CD47 from lysates of primary macrophages, but treatment with 0.1–100 ng/ml of TSP1 dose-dependently inhibited the association between CD14 and CD47 (Fig. 5B). The CD47-binding domain of TSP1 was sufficient to disrupt the interaction between CD14 and CD47 because CD14 association with CD47 decreased markedly in the presence of 2.2 pM E123CaG1, but not in the presence of equimolar concentrations of trimeric recombinant N-terminal domain of TSP1 (NoC1) or of the central CD36-binding domain 3TSR (Fig. 5C).

Signal transduction downstream of CD14/TLR4 results in NFκB-dependent transcription of target genes including IL-1β^48^. We assessed NFκB/AP-1-dependent transcriptional activity using the reporter cell line THP1-XBlue. Addition of TSP1 dose-dependently decreased the NFκB/AP-1 reporter activity induced by LPS (Fig. 5D). Since these cells are known to be only mildly responsive to LPS stimulation, we also treated with TSP1 in the presence of Pam3CSK4, a TLR1/2 agonist that also interacts with CD14^49^. As expected, Pam3CSK4 was a strong agonist of NFκB/AP-1 activity, and TSP1 dose-dependently decreased the Pam3CSK4-stimulated NFκB/AP-1 response (Fig. 5E). Therefore, we conclude that TSP1 binds to CD47 and dissociates it from CD14, thereby decreasing NFκB/AP-1 transcriptional activity stimulated by TLR4 and TLR1/2 ligands.

### TSP1 regulates IL-1β maturation by CD47-dependent and -independent mechanisms

**Figure 6:**
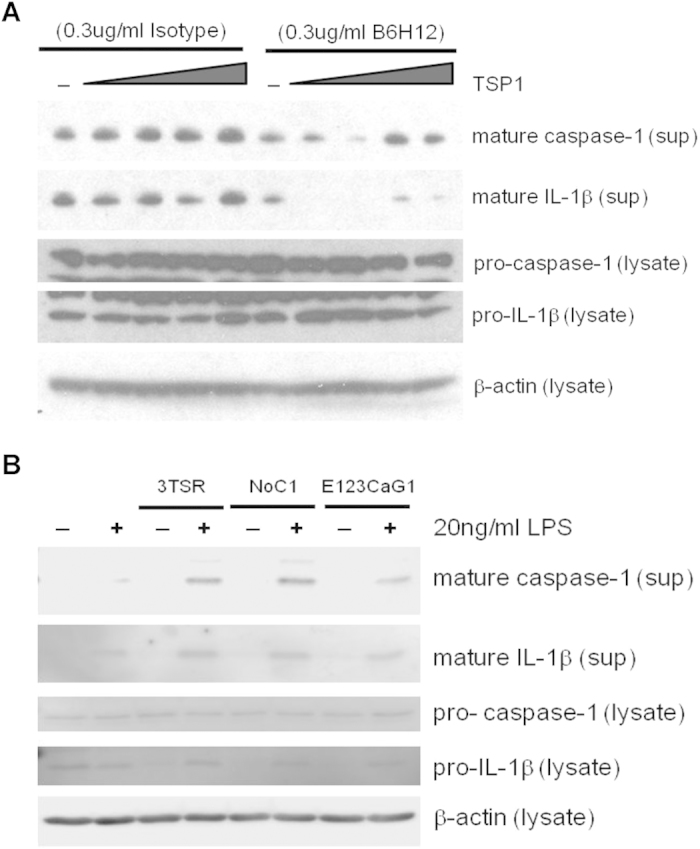
TSP1-driven IL-1β maturation is CD47-dependent. (**A**) Differentiated THP-1 cells were cultured with a mouse anti-human CD47 (B6H12) monoclonal antibody or IgG k anti-mouse isotype control for 15 min. Soluble TSP1 was added at increasing concentrations, and after 15 min, 20 ng/ml LPS was added. (**B**) THP-1 monocyte derived macrophages were treated with 22 pM of recombinant TSP1 domains: (3TSR, NoC1, or E123CaG1) for 15 min, and then either LPS or medium was added to the cells for 3 hours. sup = conditioned medium from the well following experiments; lysate = cell lysis buffer was added to wells to assess intracellular protein levels. Data shown are representative of 3 experiments performed.

To further assess the role of CD47 in maturation of pro-IL-1β we treated cells with the CD47 function-blocking antibody B6H12^47^ prior to stimulating with LPS. Mature caspase-1 and mature IL-1β were decreased when cells were treated with B6H12, whereas the isotype control had no effect (Fig. 6A). At all low concentrations of TSP1 the CD47 antibody abolished release of mature IL-1β and decreased mature caspase-1. This blocking was partially overcome at the highest TSP1 concentrations (10–100 ng/ml), where a trace of mature IL-1β was visible, and larger amounts of caspase-1 were detected. This may indicate that IL-1β maturation at higher concentrations of TSP1 is CD47-independent, whereas lower concentrations of TSP1 drive IL-1β maturation through CD47. The lower affinity of TSP1 for its receptors other than that reported for CD47^47^,^50^ suggested that enhancement of IL-1β maturation in the presence of LPS at higher concentrations of TSP1 could be mediated by other TSP1 receptors. Consistent with this hypothesis, stimulation of THP-1 monocyte-derived macrophages with LPS, in the presence of equimolar concentrations of recombinant 3TSR, NoC1, or E123CaG1 domains of TSP1 (Fig. 4H), similarly increased caspase-1 and IL-1β activation (Fig. 6B). Therefore, TSP1 signaling through several receptors, including CD47, is sufficient to promote pro-IL-1β maturation.

## Discussion

**Figure 7:**
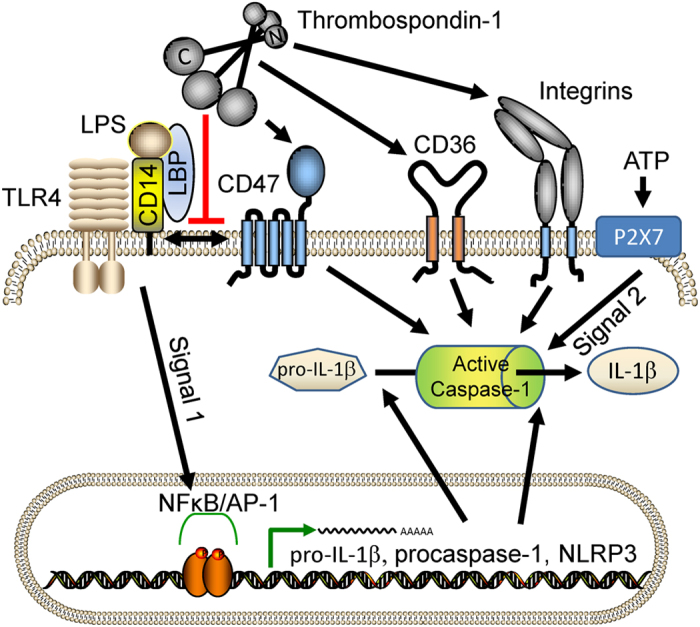
Model for TSP1 regulation of LPS-stimulated IL-1β expression and maturation in macrophages. LPS binding to CD14 and LBP initiates signal one through TLR4, which induces NFκB/AP1-dependent transcription of pro-IL-1β and pro-caspase-1. CD47 is basally complexed with CD14^36^, but binding of the C-terminal domain of TSP1 to CD47 displaces CD47 from this complex and attenuates signal one. Inflammasome-mediated maturation of pro-IL-1β and caspase-1 is induced by signal two triggered by extracellular ATP binding to purinergic receptors. CD47 signaling positively regulates signal two. TSP1 also positively regulates signal two via binding of its N-domain (NoC1), presumably to macrophage integrins^28^, and its central type 1 repeats (3TSR) to CD36^79^.

IL-1β gene expression and proteolytic activation involve two distinct signaling pathways. Although the former has been well studied since the 1980s, recognition of the role of NLRP3 and the inflammasome in signal two spurred renewed interest in the mechanism for activation of pro-IL-1β and its regulation^51^,^52^. Here we show that TSP1 signaling through CD47 modulates the transcriptional up-regulation of pro-IL-1β by LPS as well as its subsequent activation (Fig. 7). CD47 promotes LPS-dependent transcription of IL-1β, NLRP3, and caspase-1 mRNAs, but ligation of CD47 by TSP1 inhibits this signal one-dependent inflammatory response by disrupting the interaction between CD14 and CD47. Macrophages expressing CD47 correspondingly secrete higher levels of TNFα and total IL-1β. CD47 also plays a role in signal two-mediated production of mature IL-1β. LPS plus ATP stimulated less maturation of IL-1β and capase-1 in the absence of CD47. TSP1 partially regulates IL-1β maturation through CD47, but domains of TSP1 that interact with integrins and CD36 also enhance IL-1β maturation. The latter is consistent with a previous report that 22 nM (10 μg/ml) TSP1 stimulates CD36-dependent NFκB activation and TNFα expression in macrophages through TLR4^30^. Thus, regulation of signal 2-dependent maturation by TSP1 differs from signal one-dependent inhibition of pro-IL-1β mRNA induction. Signal one is inhibited by the CD47-binding domain of TSP1 (E123CaG1) but not by the CD36-binding type 1 repeats.

Our evidence that CD47 regulates both transcriptional induction of pro-IL-1β and activation of the pro-inflammatory cytokine is consistent with previous evidence that CD47 deficiency has a protective role in an LPS-induced lung injury model^53^. LPS-treated or *E. coli-*infected *cd47*^−/−^ mice had reduced levels of pro-inflammatory cytokines and neutrophil infiltration and, therefore, were protected against lung injury during infection.

Our data also suggest that CD47 deficiency protects mice against LPS-dependent pyroptosis. Pyroptosis is a pro-inflammatory process that results in elevated levels of IL-1β due to plasma-membrane rupture^43^. LPS and ATP treatment increased cell death in parallel with maturation of pro-caspase-1 in wild type cells, but CD47 deficient cells are more resistant to pyroptosis.

Intact TSP1 at physiological 10-100 pM concentrations^45^,^46^ is sufficient to induce CD47 signaling, but insufficient to signal through CD36, which requires greater than 1 nM TSP1^47^. Physiological concentrations of TSP1 inhibited signal 1-dependent activation of NFκB by upstream disruption of the constitutive association between CD47 and the LPS co-receptor CD14. Inhibition of signal one by TSP1 is CD47-specific in that the CD36-binding domain and the N-terminal LRP1- and integrin-binding domain of TSP1 were inactive. Therefore, the elevated TSP1 expression characteristic of stress or injury has proinflammatory effects in macrophages through several of its receptors, but its anti-inflammatory activity at physiological levels is specifically mediated by CD47.

Our data indicates that TSP1 is an upstream inhibitor of the NFκB/AP-1-dependent induction of IL-1β mRNA. In addition to LPS/TLR4-dependent activation, the reporter cell line used for these assays has a stronger response to TLR2 agonists, and TSP1 dose-dependently inhibited reporter activity induced by the TLR1/2 agonist Pam3CSK4. Given that both these PAMPs require that TLR co-receptor CD14 to induce NFκB/AP-1 activation^49^,^54^, we considered the possibility that TSP1 through CD47 might perturb the function of CD14. We confirmed that CD47 constitutively interacts with CD14^36^, but TSP1 treatment resulted in dissociation of CD14 from CD47. Thus, we propose that TSP1 ligation of CD47, by dissociating CD14 from CD47, prevents PAMP-induced signaling via TLR2 or TLR4 to activate NFκB/AP-1, which causes a decrease in the transcription and total protein level of pro-IL-1β (Fig. 7). The decreased expression of targets of this pathway in *cd47*^−/−^ BMDMs suggests that the constitutive interaction of CD47 with CD14 is necessary for its optimal function in PAMP signaling through these TLRs. Therefore, CD47 could be a molecular switch between TLR-independent and –dependent signaling pathways. LPS perturbs CD14-CD47 association^36^, and TSP1-CD47 ligation inhibits TLR-dependent NFκB/AP-1 transcriptional induction (Fig. 5). We therefore hypothesize that TSP1-CD47 ligation promotes CD14-dependent TLR-independent signaling pathways that result in an increased type I interferon response^55^.

Inflammasome activation can mediate the maturation of pro-IL-1β in response to signal two^42^. Although we have shown that in the presence of LPS and exogenous TSP1, NLRP3 mRNA is significantly increased, we cannot conclude that this mechanism is inflammasome dependent^56^. This study has focused on downstream effects of TSP1 on macrophage inflammatory signaling, but others have reported that peritoneal macrophages and THP1 cells secrete abundant amounts of endogenous TSP1 when stimulated with LPS^18^,^57^. This suggests that TSP1 is an autocrine regulator of macrophage inflammatory responses. Future studies should examine the temporal dependence of LPS regulation of secreted TSP1, total secreted pro-IL-1β and mature IL-1β to determine whether TSP1 limits the degree of IL-1β response or acts as a negative feedback pathway to limit the duration of the inflammatory cascade. Furthermore, our data suggests that CD47-deficient and NLRP3-deficient mice may exhibit different kinetics of IL-1β induction *in vivo*.

Our data provide a broader context for understanding the functions of different TSP1 receptors expressed by macrophages. TSP1 plays a complex role in macrophage IL-1β responses that involves CD47 and other TSP1 receptors in addition to its known activation of immunosuppressive TGF-β signaling^58^,^59^. CD47 promotes LPS-dependent IL-1β mRNA expression and ATP-dependent pro-IL-1β maturation. In contrast, TSP1 inhibits CD47-dependent IL-1β transcription, most likely through CD14. The interplay of these opposing signals could explain the biphasic responses of IL-1β mRNA levels and mature IL-1β to increasing concentrations of TSP1. CD47-mediated effects occur at physiological concentrations of TSP1 that circulate in blood plasma^46^. Because TSP1 expression is induced in response to inflammation by macrophages and other cell types and may itself be a target of signal two^16^,^57^, we propose that TSP1 is a homeostatic buffer in response to LPS stimulation.

These direct effects of TSP1 through CD47 in macrophages must be considered in the broader context that TSP1/CD47 signaling also controls adaptive immunity via inhibiting T-cell activation and differentiation^12^,^60^,^61^,^62^,^63^,^64^. TSP1 ligation of CD47 further controls immune responses by regulating maturation and trafficking of dendritic cells^65^,^66^,^32^,^67^. These TSP1/CD47 signaling pathways have pathophysiological consequences for survival of immune challenges including bacterial and fungal infections as well as in anti-tumor immunity^12^,^24^,^28^,^32^,^53^,^68^.

## Methods

### Stimulation Reagents

Adenosine 5-triphosphate disodium salt hydrate (ATP) and phorbol 12-myristate 13-acetate (PMA) were from Sigma. RPMI 1640 Medium, penicillin-streptomycin (Pen-Strep), L-glutamine (L-Gln), Versene and Opti-MEM I were from Life Technologies. Ultrapure lipopolysaccharide (LPS) and Pam3CSK4 were from InvivoGen. Fetal bovine serum (FBS) was from Gemini BioProducts. Human TSP1 was purified from platelets obtained from the National Institutes of Health Department of Transfusion Medicine, as previously described^69^. Recombinant TSP1 domains comprising the type 1 repeats (3TSR), N-terminal, oligomerization and von Willebrand C domains (NoC1), and EGF repeats, calcium-binding repeats and C-terminal domain (E123CaG1) were kind gifts from Dr. Jack Lawler^70^ and Dr. Dean Mosher^71^,^72^, respectively.

### Animals

*cd47*^−/−^ and *thbs1*^−/−^ mice were purchased from Jackson Laboratories and backcrossed to a pure C57Bl/6 background before use. Wild type C57Bl/6 mice were purchased from Charles River Laboratories under contract from the National Cancer Institute and maintained in the same vivarium for at least one month to ensure similar microflora. Animals were age and sex-matched. All experiments were performed in an accredited facility according to the NIH guidelines under protocols LP-012 and LP-022 approved by the National Cancer Institute Animal Care and Use Committee.

### BMDM Cultures and Stimulation

To make primary bone marrow-derived macrophages (BMDMs), mice were sedated using isoflurane and euthanized with cervical dislocation. Whole hind legs were removed, cleaned of all tissue and sterilized with 70% ethanol. Bones were flushed with RMPI 1640 supplemented with L-Gln. Red blood cells were lysed with ACK Lysis Buffer. Cells were plated and differentiated into macrophages using 30% L929 conditioned medium in RPMI supplemented with L-Gln, Pen-Strep, and 10% FBS as described previously^73^, L929 cells were a kind gift from Dr. Alan Sher, NIH. BMDMs were lifted using EDTA, counted and plated.

Pairs of wild type and *thbs1*^−/−^ BMDMs were incubated with either medium or LPS for 5 h with ATP or medium added for the last hour. Pairs of wild type and *cd47*^−/−^ BMDMs were primed with soluble human TSP1 or recombinant proteins in OptiMEM for 15–20 min. Cells were then treated with either medium or LPS for 3 h. ATP was added for the last half hour when indicated.

### Human Cell Cultures and Stimulations

THP-1 cells were purchased from ATCC and cultured in the presence of RMPI 1640 with 10% FBS, Pen-Strep, and L-Gln. THP-1 monocytes were differentiated by culturing for 48 h with 10 ng/ml PMA^74^.

Buffy coats or elutriated monocytes were obtained from healthy anonymous donors at the National Institutes of Health Blood Bank. Lymphocytes from buffy coats were isolated by density-gradient centrifugation using Ficoll Hypaque (GE Healthcare) and were washed with DPBS. Residual red blood cells were lysed with ACK Lysis Buffer. Cells were plated and differentiated in RPMI 1640 medium in the presence of 2% AB Human Serum (Valley Biomedical Inc.), Pen-Strep, L-Gln and 0.5 μg/ml M-CSF (PeproTech) for 6–7 d. Cells were lifted with Versene, counted, and plated. Cells were allowed to adhere for at least 1.5 h, washed, and as indicated stimulated in OptiMEM.

For both differentiated human THP-1 cells and primary monocyte derived macrophages, medium alone, soluble human TSP1 or recombinant TSP1 protein was added for 15–20 min. LPS was added for 3 h with or without ATP for the last half hour.

### ELISA

Cell culture supernatants were collected and stored at −20 °C. Human IL-1β, mouse IL-1β, and mouse TNFα protein concentrations were analyzed using human IL-1 beta/IL-1F2 DuoSet, mouse IL-1 beta/IL-1F2 DuoSet and mouse TNF-alpha kits, respectively, in accordance with the manufacturer’s published instructions (R&D Systems). Color development and reaction were terminated at the same colorimetric density for each plate using an Epoch plate reader (BioTek) and the Gen5 software. Protein concentrations were calculated using a standard curve respective to each plate.

### LDH Cytotoxicity assay

Supernatants were collected from wells following stimulation. After analysis for IL-1β and/or TNFα concentration, supernatants were incubated with an equal volume of LDH assay mix. The assay was stopped as indicated by the manufacturer’s instructions (CytoTox 96 Non-Radioactive Cytotoxicity Assay Kit, Promega). Absorbance was measured using an Epoch plate reader and Gen5 software. Kill curve and BMDM viability was calculated.

### RNA extraction and Real Time PCR

TRIzol (Life Technologies) was added to cells following stimulation experiments. Lysates and RNA were extracted according the manufacturer’s instructions. RNA was further purified using sodium acetate and 100% ethanol, then quantified with a Nanodrop 2000. cDNA was synthesized according to the manufacturer’s instructions using the SuperScript II Reverse Transcriptase Kit (Life Technologies). Quantitative real-time PCR was done using the SYBR Green kit (Thermo) and primer sets for hypoxanthine phosphoribosyltransferase, NLRP3, caspase-1, and IL-1β (Table I)^75^,^76^. Results were calculated using the ΔCt method^77^ and normalized to hypoxanthine phosphoribosyltransferase expression.

### SEAP Reporter Assay

THP1-XBlue cells (InvivoGen) were cultured as described for wild type THP-1 cells. However Zeocin (InvivoGen) was added every third passage to maintain selection. Cells were plated and differentiated with 10 ng/ml PMA for 48 h in 96-well plates. Cells were washed with DPBS, and TSP1 was used to prime the cells for 15–20 min in OptiMEM. LPS or medium alone was added for 3 h. Sterile QUANTI-Blue was added to each well, and cells were incubated at 37 °C in 5% CO_2_ for 2 h. Assays were read using the Epoch plate reader and Gen5 software.

### Immunoprecipitation

NP-40 lysis buffer (50 mM Tris pH 8.0, 150 mM NaCl, 1%NP-40, with Complete Protease Inhibitor Cocktail (Roche), sodium orthovanadate, and phenylmethyl sulfonyl fluoride) was added to monocyte derived macrophages. Cell lysates were incubated at 4 °C, collected, and spun at 4 °C to pellet cell debris. Cell lysates were transferred to a new microcentrifuge tube and pre-cleared with Protein-G Beads (Life Technologies). Monoclonal mouse anti-human CD47 monoclonal antibody (B6H12; Abcam) or LEAF purified monoclonal mouse IgG1 isotype κ control antibody (Clone MG1-45; Biolegend) was added to the lysates for 1 hr, rotating at 4 °C. Protein G beads were added, and samples were rotated at 4 °C overnight. Beads were immobilized using a magnet, and the supernatant was removed. Supernatants were combined with LDS sample buffer. Conditioned beads were washed with DPBS containing 0.1% Tween-20, and protein was eluted using sample loading buffer (Quality Biological Inc. or Thermo Scientific) and by heating at 95 °C. Supernatant and eluted bead-bound protein was analyzed using Western blotting.

### Western Blotting

Cell culture supernatants were collected into microcentrifuge tubes and stored at 4 °C for up to 1 wk. Protein from conditioned supernatants was prepared using methanol-chloroform extraction as previously described^78^. Protein was resolubilized in SDS-PAGE reducing sample loading buffer (Thermo Scientific).

Cell lysates were made by adding NP-40 lysis buffer to adherent cells. Lysates were centrifuged to remove insoluble debris, and equal volumes of lysate were transferred to clean microcentrifuge tubes containing SDS-PAGE reducing sample loading buffer.

All samples were heated at 95 °C for 5 min and cooled on ice. Proteins were separated using Tris-glycine gels and transferred to Hybond-P polyvinylidene difluoride (PVDF) membranes (GE Healthcare). Membranes were immunoblotted with polyclonal rabbit anti-mouse caspase-1 p10 (sc-514; Santa Cruz Biotechnology), monoclonal rabbit anti-human caspase-1 p20 (D7F10, Cell Signaling Technologies), polyclonal goat anti-mouse IL-1β (AF-401-NA; R&D Systems), polyclonal rabbit anti-human IL-1β (2022, Cell Signaling Technologies), monoclonal mouse anti-α-tubulin (clone DM 1A; Sigma), mouse anti-β-actin (Sigma), or monoclonal mouse anti-human CD14 (clone 134620, R&D Systems). All primary antibodies from a rabbit were detected using anti-rabbit IgG conjugated to HRP (R&D Systems), from a goat by using anti-goat conjugated to HRP (R&D Systems), and from a mouse using anti-mouse IgG conjugated to HRP (Kirkegaard & Perry Laboratories, Inc.). Membranes were incubated with West Pico, West Dura, or West Femto chemiluminescent substrate (Pierce). As needed, PVDF membranes were stripped with ReBlot Plus Mild Antibody Stripping Solution (Millipore) and reprobed with new primary and secondary antibody pairs.

### Statistics

Statistical analysis was performed with GraphPad Software and analyzed using either ANOVA or parametric Student’s t-test, as appropriate. If standard deviations were unequal as measured by Student’s t-test, Welch’s correction was applied. Methodology used in each experiment is indicated in the legends of the respective figures. Values with p ≤ 0.05 were considered statistically significant. All significance is indicated in the figure legends.

## Additional Information

**How to cite this article**: Stein, E. V. *et al.* Secreted Thrombospondin-1 Regulates Macrophage Interleukin-1β Production and Activation through CD47. *Sci. Rep.* **6**, 19684; doi: 10.1038/srep19684 (2016).
